# The impact of HBV, HCV, or syphilis infections on embryo and pregnancy outcomes in couples undergoing IVF treatment: a matched cohort study

**DOI:** 10.1093/hropen/hoaf015

**Published:** 2025-03-18

**Authors:** Fang Liu, Zheng Wang, Ying Song, Tian Tian, Rong Li, Jie Qiao, Shuo Huang, Yuanyuan Wang

**Affiliations:** Department of Obstetrics and Gynecology, Center for Reproductive Medicine, Peking University Third Hospital, Beijing, The People’s Republic of China; National Clinical Research Center for Obstetrics and Gynecology, Peking University Third Hospital, Beijing, The People’s Republic of China; State Key Laboratory of Female Fertility Promotion, Department of Obstetrics and Gynecology, Peking University Third Hospital, Beijing, The People’s Republic of China; Key Laboratory of Assisted Reproduction, Peking University, Ministry of Education, Beijing, The People’s Republic of China; Beijing Key Laboratory of Reproductive Endocrinology and Assisted Reproductive Technology, Peking University Third Hospital, Beijing, The People’s Republic of China; Department of Obstetrics and Gynecology, Center for Reproductive Medicine, Peking University Third Hospital, Beijing, The People’s Republic of China; National Clinical Research Center for Obstetrics and Gynecology, Peking University Third Hospital, Beijing, The People’s Republic of China; State Key Laboratory of Female Fertility Promotion, Department of Obstetrics and Gynecology, Peking University Third Hospital, Beijing, The People’s Republic of China; Key Laboratory of Assisted Reproduction, Peking University, Ministry of Education, Beijing, The People’s Republic of China; Beijing Key Laboratory of Reproductive Endocrinology and Assisted Reproductive Technology, Peking University Third Hospital, Beijing, The People’s Republic of China; Department of Obstetrics and Gynecology, Center for Reproductive Medicine, Peking University Third Hospital, Beijing, The People’s Republic of China; National Clinical Research Center for Obstetrics and Gynecology, Peking University Third Hospital, Beijing, The People’s Republic of China; State Key Laboratory of Female Fertility Promotion, Department of Obstetrics and Gynecology, Peking University Third Hospital, Beijing, The People’s Republic of China; Key Laboratory of Assisted Reproduction, Peking University, Ministry of Education, Beijing, The People’s Republic of China; Beijing Key Laboratory of Reproductive Endocrinology and Assisted Reproductive Technology, Peking University Third Hospital, Beijing, The People’s Republic of China; Department of Obstetrics and Gynecology, Center for Reproductive Medicine, Peking University Third Hospital, Beijing, The People’s Republic of China; National Clinical Research Center for Obstetrics and Gynecology, Peking University Third Hospital, Beijing, The People’s Republic of China; State Key Laboratory of Female Fertility Promotion, Department of Obstetrics and Gynecology, Peking University Third Hospital, Beijing, The People’s Republic of China; Key Laboratory of Assisted Reproduction, Peking University, Ministry of Education, Beijing, The People’s Republic of China; Beijing Key Laboratory of Reproductive Endocrinology and Assisted Reproductive Technology, Peking University Third Hospital, Beijing, The People’s Republic of China; Department of Obstetrics and Gynecology, Center for Reproductive Medicine, Peking University Third Hospital, Beijing, The People’s Republic of China; National Clinical Research Center for Obstetrics and Gynecology, Peking University Third Hospital, Beijing, The People’s Republic of China; State Key Laboratory of Female Fertility Promotion, Department of Obstetrics and Gynecology, Peking University Third Hospital, Beijing, The People’s Republic of China; Key Laboratory of Assisted Reproduction, Peking University, Ministry of Education, Beijing, The People’s Republic of China; Beijing Key Laboratory of Reproductive Endocrinology and Assisted Reproductive Technology, Peking University Third Hospital, Beijing, The People’s Republic of China; Department of Obstetrics and Gynecology, Center for Reproductive Medicine, Peking University Third Hospital, Beijing, The People’s Republic of China; National Clinical Research Center for Obstetrics and Gynecology, Peking University Third Hospital, Beijing, The People’s Republic of China; State Key Laboratory of Female Fertility Promotion, Department of Obstetrics and Gynecology, Peking University Third Hospital, Beijing, The People’s Republic of China; Key Laboratory of Assisted Reproduction, Peking University, Ministry of Education, Beijing, The People’s Republic of China; Beijing Key Laboratory of Reproductive Endocrinology and Assisted Reproductive Technology, Peking University Third Hospital, Beijing, The People’s Republic of China; Department of Obstetrics and Gynecology, Center for Reproductive Medicine, Peking University Third Hospital, Beijing, The People’s Republic of China; National Clinical Research Center for Obstetrics and Gynecology, Peking University Third Hospital, Beijing, The People’s Republic of China; State Key Laboratory of Female Fertility Promotion, Department of Obstetrics and Gynecology, Peking University Third Hospital, Beijing, The People’s Republic of China; Key Laboratory of Assisted Reproduction, Peking University, Ministry of Education, Beijing, The People’s Republic of China; Beijing Key Laboratory of Reproductive Endocrinology and Assisted Reproductive Technology, Peking University Third Hospital, Beijing, The People’s Republic of China; Department of Obstetrics and Gynecology, Center for Reproductive Medicine, Peking University Third Hospital, Beijing, The People’s Republic of China; National Clinical Research Center for Obstetrics and Gynecology, Peking University Third Hospital, Beijing, The People’s Republic of China; State Key Laboratory of Female Fertility Promotion, Department of Obstetrics and Gynecology, Peking University Third Hospital, Beijing, The People’s Republic of China; Key Laboratory of Assisted Reproduction, Peking University, Ministry of Education, Beijing, The People’s Republic of China; Beijing Key Laboratory of Reproductive Endocrinology and Assisted Reproductive Technology, Peking University Third Hospital, Beijing, The People’s Republic of China

**Keywords:** HBV, HCV, syphilis, live births, IVF

## Abstract

**STUDY QUESTION:**

Do infectious diseases (hepatitis B virus [HBV], hepatitis C virus [HCV], and syphilis) impact embryo quality, pregnancy, and neonatal outcomes following a complete IVF cycle?

**SUMMARY ANSWER:**

Infections with HBV, HCV, or syphilis do not have detrimental impacts on live birth rates or neonatal outcomes in couples following a complete IVF cycle.

**WHAT IS KNOWN ALREADY:**

Maternal or paternal infections with HBV, HCV, or syphilis may decrease the clinical pregnancy rate, result in poorer embryo outcomes, and lower offspring birth weight. However, there is significant controversy regarding these effects across existing studies, highlighting the need for further research.

**STUDY DESIGN, SIZE, DURATION:**

This is a retrospective matched cohort study. Data were obtained from the clinical database of couples who underwent IVF treatment at a single academically affiliated fertility clinic from January 2011 to December 2019, with follow-up extending to December 2020. Out of 180 666 complete cycles recorded, 2443 cycles fulfilled our inclusion criteria.

**PARTICIPANTS/MATERIALS, SETTING, METHODS:**

In cycles that fulfilled our inclusion criteria, there were 1997 cycles in the HBV study group, 154 cycles in the HCV study group, and 292 cycles in the syphilis study group. Each study cycle was paired with four controls based on participant age and the timing of IVF treatment, resulting in 7988 controls for the HBV group, 616 controls for the HCV group, and 1169 controls for the syphilis group. Infections could be either single-parent or biparental. The primary outcome was live birth per complete cycle (i.e. fresh cycle plus subsequent frozen-thawed cycles). Subgroup analyses were conducted dividing cycles into maternal infection and paternal infection.

**MAIN RESULTS AND THE ROLE OF CHANCE:**

In the HBV group, pregnancy outcomes (clinical pregnancy, miscarriage, and live birth rates) and neonatal birth weight were similar to that of the controls. In the HCV group, no significant differences from the controls were observed except for a lower clinical pregnancy rate in the study group (36.4% vs 42.2%, adjusted β and 95% CI: 0.62 [0.39–0.96]). Similarly, no significant differences were found in pregnancy or neonatal outcomes between the syphilis group and the control group. As for subgroup analyses, the male-only HBV infection subgroup showed a higher miscarriage rate in the study group than in the control group (22.5% vs 17.7%, adjusted β and 95% CI: 1.56 [1.07–2.28]). For the HCV and syphilis subgroups, none of the outcomes showed significant differences between either the female-only infection or male-only infection subgroups and the controls.

**LIMITATIONS, REASONS FOR CAUTION:**

Although potential confounders were considered and adjusted for, residual bias may still exist due to the study design. The inclusion of participants solely from a single center limited the generalizability of our findings to a broader context.

**WIDER IMPLICATIONS OF THE FINDINGS:**

We presented a comprehensive overview of the impact of prevalent infectious diseases on IVF outcomes, hoping to address uncertainties surrounding the decisions of couples infected with these diseases and to assist in preventing adverse reproductive outcomes in clinical practice.

**STUDY FUNDING/COMPETING INTEREST(S):**

This study was supported by the National Natural Science Foundation of China (82204052), the National Key R&D Program of China (2022YFC2705305), and the Clinical key project of Peking University Third Hospital (BYSYZD2023007). The authors declare no competing interests.

**TRIAL REGISTRATION NUMBER:**

N/A.

WHAT DOES THIS MEAN FOR PATIENTS? Many couples worldwide face common infectious diseases, such as hepatitis and syphilis, and grapple with concerns when using IVF to achieve their desire for childbirth. Despite numerous previous studies, there is contradictory evidence regarding whether these infectious diseases have detrimental effects on embryo quality, pregnancy, or neonatal outcomes after IVF treatment compared to couples unaffected by these diseases, while outcomes for serodiscordant couples (where one partner is infected while the other is not) can vary as well. Considering the notably high prevalence of hepatitis B virus (HBV) followed by hepatitis C virus (HCV) and syphilis in China, the impact of these infectious diseases on the outcome of IVF has become a major concern for the public.

In this study, we investigated embryo, pregnancy, and neonatal outcomes in women who underwent IVF after a verified infection of HBV, HCV, or syphilis. We found that there were no significant differences in pregnancy and neonatal outcomes among HBV, HCV, or syphilis groups and their respective controls, except for a decreased clinical pregnancy rate in the HCV study group compared to controls. Therefore, we strongly advocate that HBV, HCV, and syphilis infections should not be considered as deterrents for couples seeking fertility treatment. In situations where transmission is believed to be adequately blocked, couples affected by these diseases should be encouraged to use assisted reproductive technologies normally.

## Introduction

Common infectious diseases, exemplified by hepatitis and syphilis, remain significant public health challenges characterized by high prevalence, morbidity, and mortality rates. According to the World Health Organization, in 2019, ∼295.9 million (3.8%) individuals globally were living with hepatitis B virus (HBV) infection, while 57.8 million (0.8%) were living with hepatitis C virus (HCV) infection (Cui *et al.*, 2023). The annual incidence of syphilis worldwide is around 6.3 million cases, with an estimated increase to 7 million new cases by 2020 (Xiong *et al.*, 2023). In China, the prevalence of the HBs antigen (Ag) was recorded at 3.0% in 2016 (Liu *et al.*, 2023), with an estimated 93 million people having been exposed to the HBV virus, resulting in the highest rate of HBV infection globally (Schweitzer *et al.*, 2015). The prevalence of anti-HCV antibodies was comparatively low at 0.58% in 2015 on a global scale (Gao *et al.*, 2019). The estimated prevalence of syphilis has been rising since 2005, with rates reported at 0.26% for women of reproductive age (Korenromp *et al.*, 2020). Given the vast population base, these diseases pose significant public health challenges.

Infertility is another pressing global health issue affecting millions of couples, with rates varying worldwide. In China, among couples of reproductive age, one in four is affected by infertility, compared to a global average of 15% (Tatum, 2020). In addition to the high infertility rate, the discontinuation of the one-child policy in 2015 and societal changes, such as an increase in maternal age at delivery, have contributed to an increased demand for infertility treatments. Importantly, as infectious diseases have become more manageable chronic conditions due to the development of effective antiviral therapies, IVF has emerged as a common practice to assist those couples infected with HBV, HCV, or syphilis in achieving their desire for childbirth (Bourdon *et al.*, 2021).

However, it remains unclear whether these infectious diseases have detrimental impacts on IVF treatment on pregnancy and neonatal outcomes compared to couples who are not affected. The associations of HBV infection with embryo quality and pregnancy outcomes have been explored by several studies from various perspectives. For example, one meta-analysis reported that HBV infection was not significantly associated with decreased fertility rates (Farsimadan *et al.*, 2021). Another meta-analysis (Xiong *et al.*, 2022) investigating the impact of biparental HBV infection on pregnancy outcomes after ART reported that maternal HBV infection was not associated with reductions in clinical pregnancy rate and live birth rate, whereas paternal HBV infection likely decreased the clinical pregnancy rate. This finding was confirmed by a subsequent retrospective cohort study conducted in China, which included 3965 infertile couples (Zhu *et al.*, 2024). However, it has been challenged by a recently published study, finding that despite decreased sperm parameters in couples with HBV-infected males undergoing IVF, there was no impact on subsequent embryos or pregnancy outcomes (Meng *et al.*, 2024). In terms of the impact of HCV infection, a cohort study with 42 IVF cycles in HCV-seropositive women and 84 matched control cycles found that HCV-seropositive women had significantly fewer available embryos compared to controls. However, embryo morphologic features, the number of transferred embryos, and rates of implantation and pregnancy were similar for both HCV-seropositive women and controls (Englert *et al.*, 2007). Regarding syphilis, a study conducted by our team a decade ago, involving 482 couples, found that the syphilis infection status did not affect embryo transfer (ET) outcomes, including clinical pregnancy rate and neonatal birth weight (Lin *et al.*, 2014). In contrast, one study yielded opposite conclusions, indicating a decreased clinical pregnancy rate and lower offspring birth weight following syphilis infection in couples undergoing IVF treatment (Wang *et al.*, 2015).

Limitations such as small sample sizes in single-center studies, population heterogeneity, absence of gender-specific analysis, and lack of cumulative live birth analysis have restricted the robustness of conclusions drawn from existing research. Thus, the ongoing debate surrounding the risk of infectious diseases in IVF treatment necessitates further discussion. To address these gaps, we conducted a large-scale retrospective matched cohort study. Our objective was to evaluate the impact of HBV, HCV, or syphilis infection on pregnancy and neonatal outcomes in couples undergoing IVF treatment. Through this comprehensive assessment, we aimed to determine the safety and efficacy of IVF procedures for couples with infections of HBV, HCV, or syphilis, ultimately providing clinical guidance for couples facing these challenges.

## Materials and methods

### Study design

This is a retrospective matched cohort study. The study was approved by the Ethics Committee of Peking University Third Hospital (No. IRB00006761-M2020004) and conducted in accordance with the local legislation and institutional requirements. Written informed consent for participation was waived because this was a secondary data analysis with no personally identifiable information.

### Study population

Data were obtained from the clinical database of couples who underwent IVF treatment at the Reproductive Center of Peking University Third Hospital from January 2011 to December 2019, with follow-up extending to December 2020. Serological screening for the four major infectious diseases (HBV, HCV, syphilis, and HIV) is routinely conducted before couples undergo IVF treatment within a 6-month timeframe. These indicators are measured in plasma using an electro-chemiluminescence immunoassay (Roche Diagnostic, Mannheim, Germany).

Couples with documented infection history as per medical records or confirmed current infection through laboratory serological results were eligible for inclusion in this study. Specifically, the HBV study group included couples who were HBsAg positive, with or without HBeAg, HBcAb, and HBeAb positive markers, while the HCV study group comprised those who have tested positive for HCV RNA. Both HBV and HCV infections required standardized treatment for the conditions and necessitated the patients to have normal liver function to undergo IVF treatment. For the syphilis group, couples testing positive for the *Treponema pallidum* (*T. pallidum*) particle agglutination assay and rapid plasma reagin (RPR) test were treated with benzathine penicillin G. Patients with negative RPR test results or RPR titers stable and less than or equal to 1:4 may undergo IVF treatment. Infections could be either single-parent or biparental. Exclusion criteria for this study encompassed couples opting for preimplantation genetic testing (PGT) due to chromosomal structural rearrangements (PGT-SR) or monogenic/single gene defects (PGT-M) and those with incomplete laboratory data. Each study group (HBV, HCV, or syphilis) was paired with a control group, matched for age and treatment year at a 1:4 ratio, separately. We performed Mahalanobis distance matching between the infected and non-infected groups by using Python. We calculated the Mahalanobis distance between each non-infected subject and the infected subjects based on age and treatment year, selecting the four records with the smallest distance. If there were more than one non-infected subject with the same Mahalanobis distance, they were selected at random. A without-replacement matching method was employed, so the control subjects are mutually exclusive among the disease groups. The code for selecting the controls is completely blinded to the outcome measures. Since the four records with the smallest Mahalanobis distance are selected, and the number of non-infected subjects is large, there was no issue of an inadequate number of controls.

### IVF treatment

The standard protocol for IVF treatment has been previously described (Xie *et al.*, 2023). In brief, various ovarian stimulation techniques and initial doses of gonadotrophin (Gn) were utilized. Follicular growth was monitored via ultrasound, and upon the maturation of at least two dominant follicles (>18 mm diameter), HCG was administered. Oocyte retrieval occurred ∼34- to 38-h post-HCG injection. Notably, to reduce the likelihood of disease transmission and minimize the risk of infection, both laboratory and clinical procedures scheduled patients with infectious diseases to be attended to last. The oocytes were then utilized for IVF or ICSI based on sperm quality, typically within 4 h of retrieval. Women undergoing ET received luteal support consisting of daily doses of 90 mg progesterone sustained-release vaginal gel (Merck, Darmstadt, Germany) and 20 mg dydrogesterone (Abbott, Hoofddorp, the Netherlands). Any surplus embryos meeting quality standards were cryopreserved. In some cases, embryo freezing was chosen over immediate transfer. For frozen-thawed cycles, endometrial preparation followed established protocols utilizing one of three methods: artificial cycle, natural cycle, or stimulation cycle. Natural cycles were typically chosen for women with regular menstruation, while those with irregular menstruation and ovulation disorders were advised on either the artificial or stimulation cycle. Corpus luteum support during frozen-thawed cycles mirrored that of fresh ET cycles.

### Outcome measures

The primary outcome in this analysis was live birth per complete cycle (i.e. fresh cycle plus subsequent frozen-thawed cycles). Live birth was defined as any number of newborns, after 20 weeks gestation, that exhibit any sign of life (Duffy *et al.*, 2020). Secondary outcomes were embryo quality and pregnancy and neonatal outcomes. Embryo quality included the number of oocytes retrieved, inseminated/injected, fertilized and normally fertilized, and number of available embryos. Pregnancy outcomes were clinical pregnancy, ectopic pregnancy, miscarriage, and multiple births. Clinical pregnancy was defined as a pregnancy diagnosed by ultrasonographic visualization of one or more gestational sacs. An ectopic pregnancy occurs when fetal tissue implants in a location other than the uterus or attaches to an atypical or scarred area of the uterus. Miscarriage was defined as loss of a pregnancy after clinical pregnancy occurred. Multiple births were defined as a birth of at least two fetuses. Neonatal outcomes were gestational age at delivery, preterm birth (gestational age at delivery less than 37 weeks), and neonatal birthweight.

### Subgroup assignment

To investigate the impact of single-parent infection, subgroup analyses were conducted, dividing cycles into maternal infection and paternal infection. Due to the limited instances of co-infection, this analysis was not carried out.

### Statistical analysis

SPSS 25.0 (IBM, Armonk, NY, USA) was used to perform the analysis. A *P*-value of <0.05 was considered to indicate a statistically significant difference.

Baseline characteristics are presented as mean±SD or median with interquartile. Categorical variables are expressed as the proportions (percentages). Normality testing was conducted using histograms, normal probability plots (Q–Q plots), and the Kolmogorov–Smirnov (K–S) test. Comparison of baseline characteristics between the study and control groups was performed using the chi-square test for categorical variables and either Student’s *t*-test or Mann–Whitney *U*-test for continuous variables. To compare the outcomes between the two groups, a generalized estimation equations (GEE) model with an exchangeable correlation matrix was utilized, accounting for the dependency of cycles from the same couple. Both unadjusted and adjusted models correcting for confounders were established. Unadjusted and adjusted odds ratio (OR) and 95% CI or mean difference and 95% CI are reported. Data were adjusted for BMI, duration of infertility, infertility type, cause of infertility, antral follicle count (AFC), ovarian stimulation regimes, endometrial thickness, total dose of Gn, Gn duration, and fertilization method, as their impacts on the outcomes have been observed in previous literature or based on experience (Vaegter *et al.*, 2017).

## Results

### Study population

Out of the 180 666 complete cycles recorded at the Reproductive Center of Peking University Third Hospital between January 2011 and December 2019, 2443 cycles fulfilled our inclusion criteria. Specifically, there were 1997 cycles for the HBV study group, 154 cycles for the HCV study group, and 292 cycles for the syphilis study group. Each study cycle was paired with four controls, resulting in 7988 controls for the HBV group, 616 controls for the HCV group, and 1169 controls for the syphilis group.

### Baseline characteristics

Table 1 displays the baseline characteristics of cycles included in the study. In the HBV group, the mean age was 33.5 years, with a mean BMI of 22.4 ± 3.1 kg/m^2^ in the study group and 22.7 ± 3.6 kg/m^2^ in the control group (*P* = 0.08). Causes of infertility varied significantly between the groups (*P* = 0.01 for ovulatory disorders and *P* = 0.001 for male factors). No significant differences were observed in other parameters such as duration of infertility and total AFC. In the HCV group, the mean age was 35.5 years, with a mean BMI of 21.9 ± 2.9 kg/m^2^ in the study group and 22.7 ± 3.3 kg/m^2^ in the control group (*P* = 0.01). Infertility types differed significantly between the groups (*P* = 0.04), with a higher proportion of tubal factors in the study group (38.3% vs 25.0%). No significant differences were observed in other parameters. In the syphilis group, several factors showed significant differences between the groups, including BMI (*P* = 0.04), duration of infertility (*P* = 0.01), infertility type (*P* < 0.001), tubal factors (*P* < 0.001), and male factors (*P* = 0.04).

**Table 1.** Baseline characteristics.

	HBV N = 1997	HBV control N = 7988	*P*-value	HCV N = 154	HCV control N = 616	*P*-value	Syphilis N = 292	Syphilis control N = 1168	*P*-value
Age (years)	33.5 ± 5.2	33.5 ± 5.2	–	35.5 ± 5.3	35.5 ± 5.3	–	34.4 ± 5.6	34.4 ± 5.6	–
BMI (kg/m^2^)	22.4 ± 3.1	22.7 ± 3.6	0.08	21.9 ± 2.9	22.7 ± 3.3	**0.01**	23.1 ± 3.3	22.7 ± 3.4	**0.04**
Duration of infertility (years)	4.9 ± 3.9	4.9 ± 3.9	0.96	6.2 ± 5.7	5.6 ± 4.5	0.22	5.6 ± 4.3	4.9 ± 3.9	**0.01**
Infertility type (n)			0.46			**0.04**			**<0.001**
Primary infertility	1077 (54.0%)	4382 (54.9%)		63 (40.9%)	310 (50.3%)		88 (30.1%)	596 (51.1%)	
Secondary infertility	919 (46.0%)	3603 (45.1%)		91 (59.1%)	306 (49.7%)		204 (69.9%)	571 (48.9%)	
Cause of infertility (n)									
Tubal factors	449 (22.5%)	1817 (22.7%)	0.80	59 (38.3%)	154 (25.0%)	**<0.001**	95 (32.5%)	220 (18.8%)	**<0.001**
Ovulatory disorders	203 (10.2%)	974 (12.2%)	**0.01**	11 (7.1%)	57 (9.3%)	0.41	33 (11.3%)	142 (12.2%)	0.69
Male factors	1014 (50.8%)	3739 (46.8%)	**<0.001**	74 (48.1%)	290 (47.1%)	0.83	121 (41.4%)	561 (48.0%)	**0.04**
Total number of AFC (n)	9.5 ± 6.4	9.2 ± 6.3	0.06	8.1 ± 6.0	8.3 ± 6.1	0.75	8.5 ± 6.0	8.6 ± 6.4	0.82

Data are presented as mean±SD or proportions (percentage).

Data were compared between the study and the control group using chi-square test for categorical variables and Student’s *t*-test or Mann–Whitney *U*-test for continuous variables.

HBV, hepatitis B virus; HCV, hepatitis C virus; AFC, antral follicle count.

*P*-values in bold are significant (*P* < 0.05).

### IVF/ICSI characteristics

IVF/ICSI characteristics for HBV, HCV, and syphilis are shown in Table 2. In the HBV group, the study group yielded a statistically significant lower number of fresh ETs compared to the control group (66.1% vs 69.2%, *P* = 0.009). In the HCV group, no significant differences were observed in cycle characteristics, except for a lower total dose of Gn (2760 ± 1446 IU vs 3155 ± 1605 IU, *P* = 0.01). In the syphilis group, the study group had a significantly higher rate of antagonist usage (52.4% vs 42.3%, *P* < 0.001) and a lower rate of ICSI usage (71.6% vs 56.0%, *P* < 0.001) compared to the control group.

**Table 2.** IVF/ICSI characteristics.

	HBV N = 1997	HBV control N = 7988	*P*-value	HCV N = 154	HCV control N = 616	*P*-value	Syphilis N = 292	Syphilis control N = 1168	*P*-value
Ovarian stimulation regimes (n)			0.40			0.46			**<0.001**
Antagonist	783 (39.2%)	3122 (39.1%)		57 (37.0%)	238 (38.6%)		153 (52.4%)	494 (42.3%)	
Long or ultra-long	870 (43.6%)	3581 (44.8%)		65 (42.2%)	276 (44.8%)		114 (39.0%)	495 (42.4%)	
Others	344 (17.2%)	1285 (16.1%)		32 (20.8%)	102 (16.6%)		25 (8.6%)	179 (15.3%)	
Endometrial thickness (mm)	10.5 ± 1.7	10.5 ± 1.8	0.42	10.2 ± 1.9	10.5 ± 1.9	0.18	10.4 ± 1.9	10.4 ± 1.9	0.96
Total dose of Gn (IU)	2750 ± 1409	2840 ± 2168	0.08	2760 ± 1446	3155 ± 1605	**0.01**	2893 ± 1339	2915 ± 1406	0.81
Gn duration (days)	10.7 ± 3.0	10.9 ± 3.1	0.11	10.6 ± 3.3	11.0 ± 3.3	0.10	11.0 ± 3.0	10.8 ± 3.4	0.41
Fertilization method (n)			0.76			0.22			**<0.001**
IVF	1056 (54.1%)	4217 (54.5%)		91 (62.8%)	342 (57.2%)		199 (71.6%)	628 (56.0%)	
ICSI	895 (45.9%)	3519 (45.5%)		54 (37.2%)	256 (42.8%)		79 (28.4%)	494 (44.0%)	
Cycle cancelled (n)	18 (0.9%)	106 (1.3%)	0.12	5 (3.2%)	10 (1.6%)	0.19	8 (2.7%)	20 (1.7%)	0.52
Cycle no oocyte retrieved (n)	12 (0.6%)	75 (1.0%)	0.14	2 (1.4%)	6 (1.0%)	0.70	4 (1.4%)	17 (1.5%)	0.94
Number of oocytes retrieved (n)	11 (6, 16)	11 (6, 16)	0.11	10 (5, 16)	9 (5, 15)	0.41	10 (5, 15)	10 (5, 15)	0.99
Number of oocytes inseminated/injected (n)	9 (5, 15)	9 (5, 15)	0.13	10 (5, 14)	8 (4, 14)	0.99	9 (5, 14)	9 (4, 13)	0.50
Number of oocytes fertilized (n)	7 (3, 11)	7 (3, 11)	0.62	7 (3, 11)	6 (3, 11)	0.94	7 (3, 10)	6 (3, 11)	0.44
Number of normal fertilized embryos (2PN) (n)	5 (2, 9)	5 (2, 9)	0.71	5 (2, 9)	5 (2, 9)	0.59	5 (3, 8)	5 (2, 9)	0.86
Number of available embryos (n)	3 (2, 6)	3 (2, 6)	0.61	2 (1, 5)	3 (2, 6)	0.27	2 (2, 5)	2 (1, 5)	0.51
Fresh embryo transfer performed (n)	1321 (66.1%)	5526 (69.2%)	**0.01**	107 (69.5%)	425 (69.0%)	0.91	194 (66.4%)	731 (62.6%)	0.22
Cryopreservation performed (n)	994 (49.8%)	3979 (49.8%)	0.98	61 (39.6%)	285 (46.3%)	0.137	135 (46.2%)	567 (48.5%)	0.48

Number of total cryopreserved embryos (n)	0 (0, 5)	0 (0, 5)	0.77	0 (0, 2.5)	0 (0, 4)	0.133	0 (0, 4)	0 (0, 3)	0.66

Data are presented as mean±SD or median (Q25, Q75) or proportions (percentage).

Data were compared between the study and the control group using chi-square test for categorical variables and Student’s *t*-test or Mann–Whitney *U*-test for continuous variables.

HBV, hepatitis B virus; HCV, hepatitis C virus; Gn, gonadotrophins.

*P*-values in bold are significant (*P* < 0.05).

### Pregnancy and neonatal outcomes

Pregnancy and neonatal outcomes for HBV, HCV, and syphilis are shown in Tables 3 and 4 separately. For HBV, the pregnancy outcomes, including ectopic pregnancy, miscarriage, live births, and multiple pregnancies, were similar to those in the control group. The clinical pregnancy rate was slightly lower in the HBV group compared to the control group (45.2% vs 45.5%, unadjusted OR and 95 CI: 0.91 [0.87–0.95]), but this difference became insignificant after adjustment (adjusted OR and 95% CI: 1.12 [0.99–1.26]). Neonatal outcomes revealed no significant difference in gestational age (37.9 ± 2.3 weeks vs 38.0 ± 2.3 weeks, adjusted β and 95 CI: −0.05 [−0.32 to 0.22]) or neonatal birthweight for both singletons and twins. In the HCV group, there was a lower clinical pregnancy rate in the study group (36.4% vs 42.2%, adjusted β and 95% CI: 0.62 [0.39–0.96]). No significant differences were observed in other pregnancy or neonatal outcomes. Similarly, no significant differences were found in pregnancy or neonatal outcomes in the syphilis group.

**Table 3.** Pregnancy outcomes.

	HBV N = 1997	HBV control N = 7988	Unadjusted OR (95% CI) or β (95% CI)	Adjusted OR (95% CI) or β (95% CI)
Clinical pregnancy (n)	903 (45.2%)	3633 (45.5%)	**0.91 (0.87–0.95)**	1.12 (0.99–1.26)
Ectopic pregnancy (n)	17 (0.9%)	90 (1.1%)	0.75 (0.45–1.27)	0.75 (0.44–1.29)
Pregnancy loss (n)	170 (18.8%)	621 (17.1%)	1.07 (0.88–1.32)	1.22 (0.99–1.49)
Live births (n)	738 (37.0%)	3044 (38.1%)	0.91 (0.81–1.02)	0.89 (0.79–1.01)

	**HCV N = 154**	**HCV control N = 616**	**Unadjusted OR (95% CI) or β (95% CI)**	**Adjusted OR (95% CI) or β (95% CI)**

Clinical pregnancy (n)	56 (36.4%)	260 (42.2%)	0.92 (0.63–1.34)	**0.62 (0.39–0.96)**
Ectopic pregnancy (n)	1 (0.6%)	9 (1.5%)	NA	NA
Pregnancy loss (n)	7 (12.5%)	53 (20.4%)	0.70 (0.27–1.82)	0.77 (0.28–2.15)
Live births (n)	47 (30.5%)	208 (33.8%)	0.76 (0.49–1.18)	0.74 (0.44–1.24)

	**Syphilis N = 292**	**Syphilis control N = 1168**	**Unadjusted OR (95% CI) or β (95% CI)**	**Adjusted OR (95% CI) or β (95% CI)**

Clinical pregnancy (n)	122 (41.8%)	458 (39.2%)	1.12 (0.82–1.51)	1.24 (0.89–1.75)
Ectopic pregnancy (n)	4 (1.4%)	19 (1.6%)	0.84 (0.29–2.47)	NA
Pregnancy loss (n)	26 (21.3%)	74 (16.2%)	1.52 (0.91–2.56)	1.36 (0.76–2.43)
Live births (n)	97 (33.2%)	395 (33.8%)	0.93 (0.68–1.27)	1.07 (0.77–1.49)

Data are presented as mean±SD or median with interquartile (Q25; Q75) or proportions (percentage). Data were compared between the study and the control group using the generalized estimation equations. Data were adjusted for BMI, duration of infertility, infertility type, cause of infertility, number of total AFC, PCOS, endometrial thickness, fertilization method, day of embryo transfer, quality of the sperm, number of oocytes retrieved.

HBV, hepatitis B virus; HCV, hepatitis C virus.

*P*-values in bold are significant (*P* < 0.05).

**Table 4.** Neonatal outcomes.

	HBV N = 738	HBV control N = 3044	Unadjusted OR (95% CI) or β (95% CI)	Adjusted OR (95% CI) or β (95% CI)
Multiple births (twins)	169/738 (22.9%)	700/3044 (23.0%)	1.00 (0.82–1.21)	0.96 (0.78–1.17)
Gestational age (weeks)	37.9 ± 2.3	38.0 ± 2.3	−0.04 (−0.31 to 0.23)	−0.05 (−0.32 to 0.22)
Preterm birth of singletons	54/566 (9.5%)	174/2334 (7.5%)	1.31 (0.95–1.80)	1.38 (0.98–1.95)
Preterm birth of twins	82/165 (49.7%)	300/693 (43.3%)	1.07 (0.98–1.17)	1.06 (0.97–1.16)
Neonatal birthweight of singletons (g)	3322 ± 588.5	3331 ± 537.1	−0.11 (−0.07 to 0.05)	−8.32 (−65.06 to 48.43)
Neonatal birthweight of twins (means, g)	2552 ± 519.7	2528 ± 450.3	23.8 (−62.3 to 110)	−33.59 (−112.26 to 45.08)

	**HCV N = 47**	**HCV controlN = 207**	**Unadjusted OR (95% CI) or β (95% CI)**	**Adjusted OR (95% CI) or β (95% CI)**

Multiple births (twins)	11/47 (23.4%)	49/207 (23.6%)	0.99 (0.47–2.09)	0.87 (0.37–2.00)
Gestational age (weeks)	37.7 ± 1.7	37.8 ± 2.1	−0.25 (−0.90 to 0.39)	−0.27 (−0.89 to 0.35)
Preterm birth of singletons	7/36 (19.4%)	16/158 (10.1%)	1.10 (0.96–1.26)	1.14 (0.96–1.35)
Preterm birth of twins	5/11 (45.5%)	20/49 (40.8%)	0.83 (0.22–3.09)	0.58 (0.12–2.68)
Neonatal birthweight of singletons (g)	3159 ± 530.6	3321 ± 681.6	−178.11 (−452.79 to 96.57)	−146.23 (−390.60 to 98.14)
Neonatal birthweight of twins (means, g)	2551 ± 440.0	2423 ± 650.5	127.82 (−178.72 to 434.38)	91.03 (−172.20 to 354.26)

	**Syphilis N = 97**	**Syphilis controlN = 394**	**Unadjusted OR (95% CI) or β (95% CI)**	**Adjusted OR (95% CI) or β (95% CI)**

Multiple births (twins)	19/97 (19.6%)	82/394 (20.8%)	0.93 (0.53–1.63)	1.01 (0.92–1.12)
Gestational age (weeks)	37.8 ± 2.2	38.2 ± 1.9	−0.30 (−0.83 to 0.23)	−0.21 (−0.76 to 0.34)
Preterm birth of singletons	9/78 (11.5%)	18/312 (5.8%)	2.13 (0.92–4.95)	0.92 (0.31–2.74)
Preterm birth of twins	10/17 (58.8%)	33/77 (42.9%)	1.91 (0.66–5.53)	0.77 (0.14–4.22)
Neonatal birthweight of singletons (g)	3261 ± 671.2	3300 ± 574.8	−72.41 (−252.85 to 108.01)	−44.03 (−220.46 to 132.40)
Neonatal birthweight of twins (means, g)	2480 ± 429.8	2552 ± 375.6	−131.11 (−302.59 to 40.37)	−164.5 (−343.58 to 14.53)

Data are presented as mean±SD or median with interquartile (Q25; Q75) or proportions (percentage). Data were compared between the study and the control group using the generalized estimation equations. Data were adjusted for BMI, duration of infertility, infertility type, cause of infertility, number of total AFC, PCOS, endometrial thickness, fertilization method, day of embryo transfer, quality of the sperm, number of oocytes retrieved.

HBV, hepatitis B virus; HCV, hepatitis C virus.

### Subgroup analyses

Supplementary Table S1 presents the results of subgroup analyses (female-only infection and male-only infection) for HBV, HCV, and syphilis, respectively. In the HBV subgroup, the rate of pregnancy loss in the male-only infection subgroup was higher in the study group than in the control group (22.5% vs 17.7%, adjusted β and 95% CI: 1.56 [1.07–2.28]). No significant differences were observed between the study and control groups for other outcomes. Regarding the HCV and syphilis subgroup, none of the outcomes showed significant differences within either the female-only infection or male-only infection subgroups.

### Sensitivity analysis

We tried different GEE models with added confounders and performed a sensitivity analysis (Supplementary Table S2). Model1 included age, BMI; Model2 included BMI, duration of infertility, infertility type, cause of infertility; Model3 included BMI, duration of infertility, infertility type, cause of infertility, number of total AFC, PCOS, endometrial thickness, quality of the sperm, number of oocytes retrieved. We found that the primary outcome remained stable across all different models.

## Discussion

In this large retrospective matched cohort study, we investigated the impact of infections with HBV, HCV, or syphilis on embryo quality, as well as pregnancy and neonatal outcomes in couples undergoing IVF treatment. Our findings indicated that there were no significant differences in pregnancy and neonatal outcomes among HBV, HCV, or syphilis groups and their respective controls, except for a decreased clinical pregnancy rate in the HCV study group compared to controls, which was observed in the adjusted analysis. In addition, we performed subgroup analyses distinguishing between female and male infections. In the HBV group, the male-only infection subgroup showed a higher miscarriage rate in the study group compared to the control group. Within the HCV and syphilis groups, none of the examined outcomes showed significant differences in either the female-only infection or male-only infection subgroups.

The safety of couples undergoing IVF treatment with HBV infection has been a concern (Lutgens *et al.*, 2009). Studies have shown that HBV DNA can integrate into the genome of oocytes and subsequently be transmitted to embryos through fertilization with sperm (Hu *et al.*, 2011). This integration may induce inheritable changes, altering genetic components and causing mutations, thereby facilitating vertical HBV transmission to offspring. Consequently, it is plausible to assume that these potential adverse effects may affect the outcomes of IVF treatment. However, our analysis did not reveal significant differences in pregnancy and neonatal outcomes, including clinical pregnancy, miscarriage, live birth rate, or neonatal birthweight after adjustment, although prior to adjustment, the clinical pregnancy rate was slightly lower in the HBV group. The mechanism behind these findings remains unclear. With HBV infection, extrahepatic tissues and cells can evade immune damage due to minimal viral presence and incomplete protein expression, resulting in negligible pathological changes (Chen *et al.*, 2005), thus we assume that the impact of HBV infection on oocytes and the reproductive environment may not be sufficient to manifest differences in embryological parameters and pregnancy rates. In the male-only HBV subgroup, there was a statistically higher rate of miscarriages compared to the control group. This finding is consistent with a recent study (Zhu *et al.*, 2024) consisting of 821 couples and 821 controls, which also showed that male HBV infection increases the risk of miscarriage.

It has been noted that the pathophysiology of HCV infection encompasses a multitude of extra-hepatic changes potentially affecting other body systems (Mazzaro *et al.*, 2021). However, it remains debatable whether HCV status modifies IVF outcomes. Some studies have suggested that HCV infection might alter both the reserve of small pre-antral follicles and granulosa cell function leading to a poor ovarian response to stimulation (Englert *et al.*, 2007), which may have a negative impact on pregnancy rates. Moreover, HCV infection in males may lead to significantly higher anti-sperm antibodies than controls, which result in a reduced sperm motility (Hussein *et al.*, 2017). On the other hand, there was a study that reported that active HCV infection did not affect follicle development nor the human luteinized granulosa cells, and thus there were no adverse effect on pregnancy outcomes (Sifer *et al.*, 2002). In our analysis, we found that the clinical pregnancy rate in the HCV group was significantly lower than that in the control group, while no other pregnancy outcomes such as miscarriage or neonatal outcomes were observed between the two groups. This result is consistent with a previous study with small sample size (Pirwany *et al.*, 2004). Notably, when both male and female infections were analyzed separately, there were no significant differences between the HCV groups and the control groups. In a meta-analysis investigating female-only infection with HCV in ART outcomes, out of five eligible studies, three studies observed an adverse impact on embryo outcomes and pregnancy rates, while no differences were found by other two studies (Shaw-Jackson *et al.*, 2017). Various factors, such as different matching criteria, sample size, viral replication level, and whether male HCV infection was considered could cause heterogeneity of IVF outcomes. And to be noted, except for our study, all studies mentioned above had restricted the analysis to the first IVF cycle, which may cause a deviation from the real clinical scenario. Still, more studies are needed to explore the effect of HCV infection in pregnancy outcomes and verify our findings.

Syphilis, an enduring chronic multisystem disorder triggered by *T. pallidum*, one of humanity’s oldest known ailments, has experienced a resurgence in recent years (Tiecco *et al.*, 2021). Syphilis can lead to pelvic inflammatory diseases, causing endometrial damage. This can result in thickening of the endometrial lining, impairing its function and reducing fertility (Van Gerwen *et al.*, 2022). Moreover, untreated maternal syphilis is well-documented to correlate with adverse pregnancy outcomes (Gomez *et al.*, 2013). However, with the advent of penicillin treatment since 1943, syphilis has become a curable ailment (Workowski *et al.*, 2021). An increasing number of infertile syphilis-infected couples have turned to IVF treatment after receiving penicillin treatment. Yet, the impact of syphilis infection on IVF outcomes has not been fully evaluated. In our analysis, we found no significant differences in pregnancy or neonatal outcomes between the study and control groups (after adjustment), nor in subgroup analyses stratifying female-only and male-only infections. These findings are somehow contrasting with a previous study on the same subject, which noted lower oocyte quality (normally fertilized and normally cleaved fertilized oocytes), clinical pregnancy rates, and offspring birth weights in the syphilis group compared to controls (Wang *et al.*, 2015). A noteworthy difference between our studies is that while the previous one (Wang *et al.*, 2015) only considered the initial fresh ET, we evaluated results from all completed cycles. Assessing only one cycle of fresh ET while ignoring subsequent frozen–thawed cycles may potentially underestimate the impact of the study group on pregnancy outcomes, leading to biased conclusions. Importantly, our current findings align with a prior study by the same research group (Lin *et al.*, 2014), suggesting no deleterious effects of IVF treatment post-penicillin therapy in syphilis-infected couples compared to controls.

Attempting IVF in couples with infectious diseases is justified as infectious diseases have become more manageable chronic conditions (Bourdon *et al.*, 2021). We aimed to present a comprehensive overview of the impact of prevalent infectious diseases on IVF outcomes, hoping to address uncertainties surrounding the decision of couples infected with these diseases and assist in preventing the adverse reproductive outcomes in clinical practice. We would like to emphasize several strengths of our study. First, we have a substantial sample size for the HBV infection group, which enhances the robustness of our findings. Additionally, our study addresses a significant gap in the literature by contributing valuable evidence on the association between HCV or syphilis infection and IVF outcomes. We conducted a thorough analysis of well-characterized participants sourced from a clinical database, ensuring rigorous monitoring both during and after IVF treatment. Importantly, unlike many previous studies that only focus on outcomes from a single cycle potentially underestimating the effect, we evaluated and reported outcomes from all cycles performed. Furthermore, we accounted for within-subject effects using appropriate analytical methods. All these approaches enable us to thoroughly evaluate the efficacy and safety of infectious diseases on IVF outcomes from various perspectives. Nevertheless, there are several limitations that should be considered when interpreting the findings of this study. It is retrospective in nature, and despite our efforts to consider potential confounders and adjust them in the statistical model, residual bias may still exist. The inclusion of participants solely from a single center limited the generalizability of our findings to a broader context due to the homogeneity of the sample. Since our study is based on the cumulative live birth rate of a complete oocyte retrieval cycle, which includes both fresh and frozen ETs, we did not adjust for certain confounders, such as the type of endometrial preparation and culture conditions, as these can vary between fresh and frozen ET, even within the same oocyte retrieval cycle. Furthermore, due to the missing data of sperm quality testing in the clinical database used in this study, we failed to make an additional match based on the quality of sperm in the male-infected subgroup analysis.

In summary, we observed that HCV infection was associated with a reduction in clinical pregnancy rates when compared to controls, while male-only HBV infection exhibited an increase in miscarriage rates. However, our study suggested that infections with HBV, HCV, or syphilis did not significantly influence pregnancy and neonatal outcomes in couples undergoing IVF treatment. These insights are of considerable importance for clinical consultations with couples affected by HBV, HCV, or syphilis infections, facilitating informed decision-making concerning their reproductive health journey.

## Supplementary Material

hoaf015_Supplementary_Data

## Data Availability

The data underlying this article will be shared upon reasonable request to the corresponding authors.
