# Supplementary material for: The impact of HBV, HCV, or syphilis infections on embryo and pregnancy outcomes in couples undergoing IVF treatment: a matched cohort study
Source: Hum Reprod Open. 2025 Mar 18;2025(2):hoaf015. doi: 10.1093/hropen/hoaf015 (PMC11961197; doi:10.1093/hropen/hoaf015)
Supplement: hoaf015_Supplementary_Data [file hoaf015_supplementary_data.docx]

Supplementary Table S1. Subgroup analysis

|  | Female-only | | | Male-only | | |
| --- | --- | --- | --- | --- | --- | --- |
|  | HBV  N=1343 | HBV control  N=5372 | Adjusted OR (95% CI) | HBV  N=637 | HBV control  N=2548 | Adjusted OR (95% CI) |
| Clinical pregnancy (n) | 602/1343 (44.8%) | 2479/5372 (46.1%) | 0.87 (0.74 to 1.03) | 293/637 (46.0%) | 1121/2548 (44.0%) | 1.14 (0.89 to 1.46) |
| Ectopic pregnancy (n) | 15/1343 (1.1%) | 50/5372 (0.9%) | 1.34 (0.71 to 2.40) | 2/637 (0.3%) | 37/2548 (1.5%) | NA |
| Pregnancy loss (n) | 103/602 (17.1%) | 417/2479 (16.8%) | 0.98 (0.72 to 1.33) | 66/293 (22.5%) | 198/1121 (17.7%) | **1.56 (1.07 to 2.28)** |
| Live births (n) | 504/1343 (37.5%) | 2087/5372 (38.8%) | 0.89 (0.76 to 1.05) | 227/637 (35.6%) | 929/2548 (36.5%) | 0.93 (0.72 to 1.19) |
|  | HCV  N=113 | HCV control  N=452 | Adjusted OR (95% CI) | HCV  N=40 | HCV control  N=160 | Adjusted OR (95% CI) |
| Clinical pregnancy (n) | 43/113 (38.1%) | 197/452 (43.6%) | 0.61 (0.35 to 1.08) | 13/40 (32.5%) | 63/160 (39.4%) | 1.07 (0.47 to 2.40) |
| Ectopic pregnancy (n) | 1/113 (0.9%) | 9/452 (2.0%) | NA | 0/40 (0%) | 0/160 (0%) | NA |
| Pregnancy loss (n) | 6/43 (14.0%) | 41/197 (20.8%) | 0.81 (0.25 to 2.61) | 1/13 (7.7%) | 12/63 (19.0%) | 0.41 (0.04 to 4.05) |
| Live births (n) | 36/113 (31.9%) | 156/452 (34.5%) | 0.66 (0.35 to 1.26) | 11/40 (27.5%) | 52/160 (32.5%) | 1.16 (0.51 to 2.63) |
|  | syphilis  N=269 | syphilis control  N=1076 | Adjusted OR (95% CI) | syphilis  N=20 | syphilis control  N=80 | Adjusted OR (95% CI) |
| Clinical pregnancy (n) | 111/269 (41.3%) | 422/1076 (39.2%) | 1.25 (0.87 to 1.79) | 11/20 (55.0%) | 33/80 (41.3%) | NA |
| Ectopic pregnancy (n) | 4/269 (1.5%) | 18/1076 (1.7%) | NA | 0/20 (0%) | 1/80 (1.3%) | NA |
| Pregnancy loss (n) | 23/111 (20.7%) | 72/422 (17.1%) | 1.18 (0.63 to 2.22) | 3/11 (27.3%) | 2/33 (6.1%) | NA |
| Live births (n) | 90/269 (33.5%) | 363/1076 (33.7%) | 1.16 (0.81 to 1.67) | 7/20 (35.0%) | 29/80 (36.3%) | NA |

Data are presented as mean ± SD or median with interquartile (Q25; Q75) or proportions (percentage). Data were compared between the study and the control group using the generalized estimation equations. Data were adjusted for BMI, duration of infertility, infertility type, cause of infertility, number of total AFC, PCOS , endometrial thickness, fertilization method, day of embryo transfer, quality of the sperm, number of oocytes retrieved

P-values in bold are significant (P < 0.05).

Supplementary Table S2. Sensitivity analysis

|  | Model1 | Model2 | Model3 |
| --- | --- | --- | --- |
| HBV infection | | | |
| Clinical pregnancy | 0.70 (0.46 to 1.05) | 0.72 (0.48 to 1.07) | 0.65 (0.41 to 1.03) |
| Ectopic pregnancy | NA | NA | NA |
| Pregnancy loss | 0.76 (0.29 to 1.97) | 0.77 (0.30 to 1.94) | 1.24 (0.48 to 3.21) |
| Live births | 0.75 (0.48 to 1.18) | 0.76 (0.49 to 1.17) | 0.69 (0.42 to 1.12) |
| HCV infection | | | |
| Clinical pregnancy | 0.70 (0.48 to 1.04) | 0.72 (0.48 to 1.09) | 0.72 (0.48 to 1.07) |
| Ectopic pregnancy | NA | NA | NA |
| Pregnancy loss | 1.32 (0.51 to 3.44) | 1.20 (0.47 to 3.06) | 0.81 (0.31 to 2.10) |
| Live births | 1.33 (0.85 to 2.10) | 1.36 (0.87 to 2.11) | 1.45 (0.89 to 2.37) |
| Syphilis infection | | | |
| Clinical pregnancy | 0.88 (0.65 to 1.20) | 1.16 (0.85 to 1.58) | 1.25 (0.90 to 1.75) |
| Ectopic pregnancy (n) | 1.07 (0.35 to 3.23) | 1.06 (0.77 to 1.47) | 1.07 (0.76 to 1.50) |
| Pregnancy loss (n) | 1.466 (0.86 to 2.50) | 1.31 (0.75 to 2.28) | 1.42 (0.80 to 2.51) |
| Live births (n) | 0.96 (0.69 to 1.34) | 1.06 (0.77 to 1.47) | 1.07(0.76 to 1.50) |

Model1 included age, BMI

Model2 included BMI, duration of infertility, infertility type, cause of infertility

Model3 included BMI, duration of infertility, infertility type, cause of infertility, number of total AFC, PCOS, endometrial thickness, quality of the sperm, number of oocytes retrieved

NA not available. Due to limited account of event
